# Individuals’ preference on reading pathways influences the involvement of neural pathways in phonological learning

**DOI:** 10.3389/fpsyg.2022.1067561

**Published:** 2022-12-14

**Authors:** Jie Dong, Qingxin Yue, Aqian Li, Lala Gu, Xinqi Su, Qi Chen, Leilei Mei

**Affiliations:** ^1^Philosophy and Social Science Laboratory of Reading and Development in Children and Adolescents (South China Normal University), Ministry of Education, Guangzhou, China; ^2^School of Psychology, South China Normal University, Guangzhou, China; ^3^Guangdong Key Laboratory of Mental Health and Cognitive Science, South China Normal University, Guangzhou, China

**Keywords:** inter-individual variability, word reading, reading pathway preference, phonological learning, fMRI

## Abstract

**Introduction:**

Existing behavioral and neuroimaging studies revealed inter-individual variability in the selection of the two phonological routes in word reading. However, it is not clear how individuals’ preferred reading pathways/strategies modulate the involvement of a certain brain region for phonological learning in a new language, and consequently affect their behavioral performance on phonological access.

**Methods:**

To address this question, the present study recruited a group of native Chinese speakers to learn two sets of artificial language characters, respectively, in addressed-phonology training (i.e., whole-word mapping) and assembled-phonology training conditions (i.e., grapheme-to-phoneme mapping).

**Results:**

Behavioral results showed that the more lexical pathways participants preferred, the better they performed on newly-acquired addressed characters relative to assembled characters. More importantly, neuroimaging results showed that participants who preferred lexical pathway in phonological access show less involvement of brain regions for addressed phonology (e.g., the bilateral orbitofrontal cortex and right pars triangularis) in the processing of newly-acquired addressed characters.

**Conclusion:**

These results indicated that phonological access *via* the preferred pathway required less neural resources to achieve better behavioral performance. These above results provide direct neuroimaging evidence for the influence of reading pathway preference on phonological learning.

## Introduction

Cognitive models of word reading (e.g., the dual-route model and triangle model) have postulated two pathways in the process of transforming visual words into their phonologies (i.e., phonological access; Plaut et al., 1996; Coltheart et al., 2001; Harm and Seidenberg, 2004; Perry et al., 2007; Reynolds et al., 2011). There is one pathway that is essential for the reading aloud of nonwords and uses grapheme-phoneme correspondences (which can be called the nonlexical pathway), and there is the other pathway which is essential for the reading aloud of irregular words (which can be called the lexical pathway). It has been repeatedly found that the respective engagement of lexical and nonlexical pathways in word reading varies across different scripts and across different types of words even within a script. Specifically, logographic languages (e.g., Chinese) that do not have grapheme-to-phoneme correspondence (GPC) rules solely rely on the lexical pathway (Chen et al., 2009). In contrast, alphabetic languages mapping graphemes onto phonemes rely more on the nonlexical pathway (Paulesu et al., 2000). This is more pronounced for languages with shallow orthography (e.g., Italian) than deep orthography (e.g., English). Within the same script, reading pseudowords and unfamiliar regular words mainly depends on the nonlexical pathway, whereas reading familiar words and irregular words depends more on the lexical pathway (Coltheart et al., 2001; Cummine et al., 2013).

By contrasting materials mentioned above, neuroimaging studies have revealed that the two pathways have distinct neural substrates. Specifically, lexical and nonlexical routes, respectively, recruit brain regions in ventral (occipito-temporal) and dorsal (occipito-parietal) pathways (Borghesani et al., 2020). Meta-analyses on those studies have further suggested that the lexical route includes the left anterior fusiform gyrus, middle temporal gyrus, angular gyrus, and the ventral part of inferior frontal gyrus (Jobard et al., 2003; Mechelli et al., 2003; Taylor et al., 2013; Sliwinska et al., 2015), whereas the nonlexical pathway includes the left posterior fusiform cortex, temporoparietal cortex, and dorsal part of inferior frontal gyrus (Wilson et al., 2012; Cattinelli et al., 2013; Taylor et al., 2013; Hoffman et al., 2015; Provost et al., 2016). Consistently, by adopting a well-designed artificial language training paradigm to control for confounding factors such as visual form, phonology, semantics, and amount of learning in the contrast of verbal materials in the natural language, Mei et al. (2014, 2015) found a clear dissociation of the neural mechanisms for lexical and nonlexical pathways. Specifically, they found that the lexical pathway relied more on the left orbital frontal cortex, middle temporal gyrus, and angular gyrus, whereas the nonlexical pathway depended more on the left precentral gyrus/inferior frontal gyrus and supramarginal gyrus.

Besides the respective involvement of ventral and dorsal neural pathways in lexical and nonlexical routes at the population level (Jobard et al., 2003; Cattinelli et al., 2013; Taylor et al., 2013; Mei et al., 2014, 2015), it appears to show inter-individual variability in the selection of two phonological pathways (Pecini et al., 2008; Ihnen et al., 2013; Mei et al., 2015). For example, Ihnen et al. (2013) showed that, in contrast with heavy reliance on the nonlexical pathway in the regularization task (i.e., pronouncing words *via* GPC rules) in children, young adults recruited both lexical and nonlexical pathways. Consistently, neuroimaging studies have also found inter-individual variability in involvement of the two neural pathways corresponding to lexical and nonlexical routes (Pecini et al., 2008; Mei et al., 2015). For instance, Pecini et al. (2008) showed that most of Italian participants utilized the nonlexical route (the left inferior frontal gyrus and dorsolateral prefrontal cortex) to perform the rhyme generation/judgment task, while a minority of participants recruited both lexical (left temporo-parietal-occipital junction) and nonlexical route (left superior temporal gyrus; Démonet et al., 1992; Paulesu et al., 2000).

Although there is both behavioral and neuroimaging evidence for inter-individual variability in the selection of the two phonological routes in word reading, it is not clear how individuals’ preferred reading pathways/strategies modulate the involvement of a certain brain region for phonological access in phonological learning, and consequently affect their behavioral performance on phonological access, especially in speakers with similar language backgrounds. To address this question, the present study adopted an artificial language training paradigm which is able to clearly dissociate form-sound learning *via* the lexical or nonlexical routes after controlling for differences in materials and the amount of learning (Mei et al., 2014, 2015). To be specific, we recruited a group of native Chinese speakers to learn two sets of artificial language words, respectively, in addressed-phonology training (i.e., whole-word mapping) and assembled-phonology training conditions (i.e., grapheme-to-phoneme mapping). Following Destoky et al. (2020) method, we quantified individuals’ preferred reading pathways/strategies by calculating reading strategy index (i.e., standardized scores of irregular word reading minus those of pseudoword reading). Given that lexical and nonlexical routes, respectively, support reading of irregular words and pseudowords, the contrast between corresponding standardized scores indicates reading strategy. Multivariate pattern analysis (MVPA) was used to identify brain regions for lexical and nonlexical processing. Finally, individuals’ reading strategy indices were correlated with their differences in reading performance between addressed- and assembled-phonology training conditions and with their activation differences between the two training conditions to examine the effects of individuals’ reading pathway preference on phonological learning in a new language.

## Materials and methods

### Participants

Twenty-five native Chinese college students (mean age = 21.68 years, SD = 1.82, 19 female) were collected in this study. All participants’ native language was Chinese, and their second language was English. They started to learn English at the mean age of 7 years (SD = 2.42) and by the time of the experiment had received formal education in the English language for 13 years (SD = 2.62). Their proficiency in English was self-reported on a 5-point questionnaire, which has been widely used in previous research on bilingualism (Jamal et al., 2012; Cao et al., 2017; Kachlicka et al., 2019). The mean scores of reading, writing, dictation and oral expression in English were 4.36 (SD = 0.70), 3.96 (SD = 0.73), 3.84 (SD = 0.94) and 3.84 (SD = 0.62), respectively. Therefore, all the participants were intermediate bilinguals, and had similar proficiency levels in their second language. They were unfamiliar with the Korean language participated in this study. To estimate the number of appropriate participants needed for our design, we used the G*Power analysis and found that 17 participants are sufficient for medium effect size (i.e., 0.25) with 0.80 power in one-way repeated-measures analysis of variance (ANOVA; Cohen, 1988, 1992). All participants’ vision or corrected vision was normal and had no related psychiatric history. They were all right-handed as measured by Snyder and Harris’s handedness inventory (Snyder and Harris, 1993). The above-mentioned inclusion and exclusion criteria were established before the data collection. Before the experiment, all participants signed an informed written consent and the procedures were approved by the IRB at the South China Normal University. No part of the study procedures and analyses was pre-registered prior to the research being conducted.

### Materials

**Figure 1:**
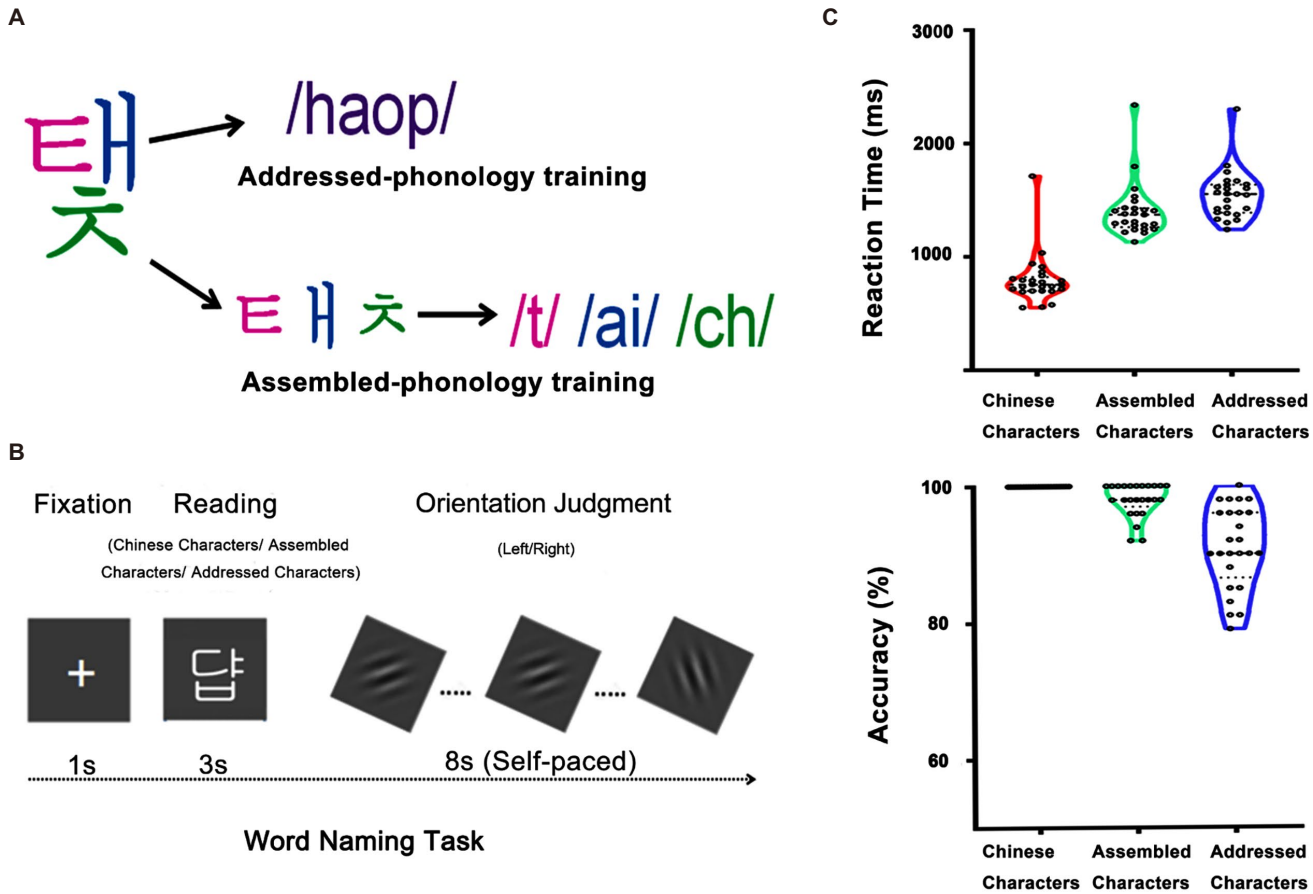
Participants were asked to learn the associations between visual forms and sounds of 32 different artificial language characters. Half were learned through addressed phonology and the other half were learned through assembled phonology **(A)**. After training, they performed one session of the word naming task during scanning and another session outside the scanner, in which they were asked to read each character aloud **(B)**. The right panels showed reaction times and accuracies of Chinese characters, assembled and addressed characters after the scan **(C)**.

One group Chinese characters and two groups of artificial language characters were used in this study. Each group consisted of 16 characters. The artificial language characters were used for learning. The Chinese characters were used as a control for learning and consequently only used in the fMRI task. All characters are presented with 226 × 151 pixels in size on a gray background screen (see Figure 1B). Chinese characters had 2–3 units (*M* = 2.1), and 6–11 strokes (*M* = 8.5). All of them were medium-frequency characters (*M* = 16.9 per million, ranging from 10/million to 30/million; Cai and Brysbaert, 2010).

The artificial language characters used in this study were constructed using 16 Hangul letters (eight consonants and eight vowels). The consonants and vowels were then divided into two matched groups. Each group consisted of four consonants and four vowels, which were used to construct 16 artificial language characters (CVC) with 3 units and 6–11 strokes. In total, 32 artificial language characters were constructed. The two groups of artificial language characters (each group had 16 characters) were assigned into the two learning conditions (i.e., the addressed-phonology and assembled-phonology learning) as described in Training Procedure blow and they did not differ in the number of strokes [the first group: *M* = 8.50; the second group: *M* = 8.25; *t*(15) = 0.37, *p* = 0.72].

The sounds of artificial language letters and characters were initially recorded from one female college students. Adobe Audition CS6 software was used to denoise the speech materials, and normalize them to the same volume and length. The sound of each character and letter lasted for 800 and 600 ms, respectively.

### Behavioral assessments

**Figure 2:**
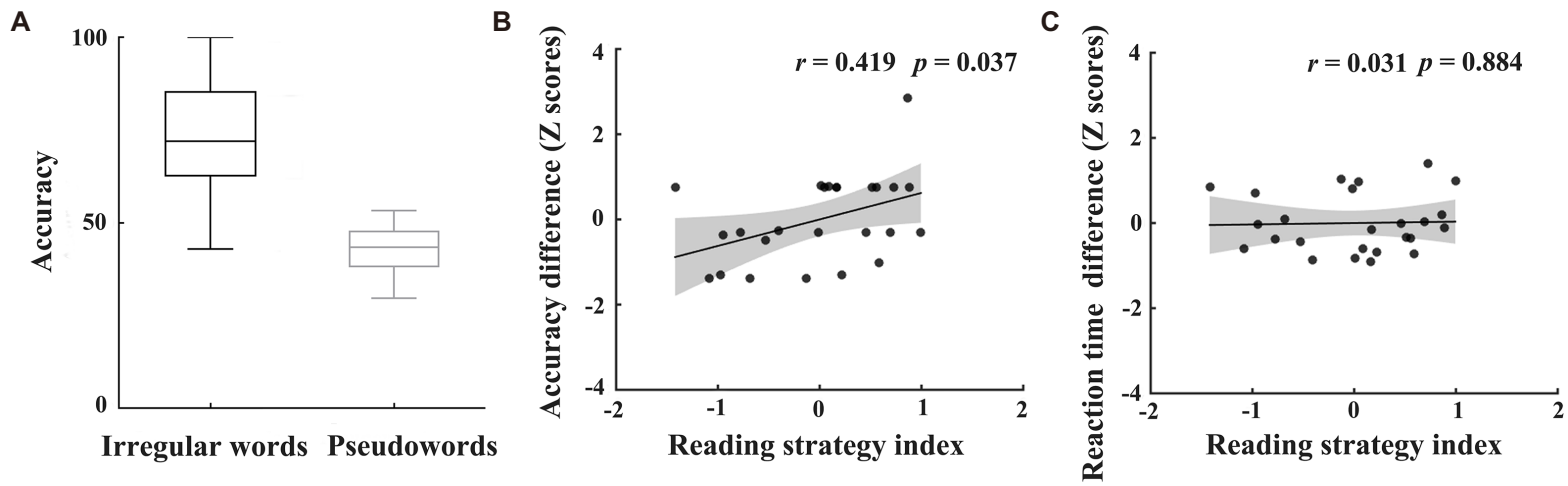
Results of individuals’ reading pathway preference. The accuracies of irregular words and regular pseudowords **(A)**. The correlations between reading strategy index and difference in accuracy **(B)** or reaction time **(C)** between assembled and addressed characters during the word naming task after fMRI scan. The values of accuracy or reaction time in panels B and C were transformed into Z scores.

To evaluate participants’ preferred routes in reading, we used two fluency tests to assess the reading fluency of the irregular words and pseudowords. The test for pseudowords was adopted from the Phonemic Decoding Efficiency subtest of TOWRE (Test of Word Reading Efficiency; Torgesen et al., 1999), which consisted of 63 phonemically regular pseudowords. The test of irregular words, consisting of 104 phonemically irregular words (e.g., whose, hour), was developed by the authors in this study. The number of irregular words was determined according to the Sight Word Efficiency subtest of TOWRE, which consisted of 104 real words. The irregular words used in this study have at least one grapheme whose phoneme is not the most common phoneme for that grapheme. Following previous studies (Berndt et al., 1987; Gontijo et al., 2003), we also quantified the pronunciation regularity by calculating the probabilities of graphemes being pronounced as their corresponding phonemes. The mean pronunciation regularity was 0.65 (SD = 0.14), suggesting that they were phonemically irregular words. In both tests, participants were asked to read words/pseudowords one by one as quickly and accurately as possible. Following the instruction of TOWRE, they were scored by using the number of correctly pronounced items within 45 s (see Figure 2A).

Given previous findings that reading irregular words and regular pseudowords, respectively, rely on the lexical pathway (depending on whole-word mapping) and nonlexical pathway (depending on grapheme-to-phoneme mapping), the difference in standardized scores between irregular words and pseudowords was used as a reading strategy index (Destoky et al., 2020). The positive reading strategy index indicates greater reliance on whole-word mapping during word reading, the negative reading strategy index indicates the tendency to use grapheme-to-phoneme mapping.

### Training procedure

All participants received artificial language training for 5 days, 1 h per day. Participants were asked to learn the associations between visual forms and sounds of 32 artificial language characters. As mentioned before, the characters were divided into two groups (each had 16 characters) for the two training conditions: addressed-phonology and assembled-phonology training (see Figure 1A). In the addressed-phonology training condition, participants were asked to associate the whole character with its sound by rote memorization. The correspondence between the characters and their original sounds was randomly shuffled to ensure that participants could not implicitly acquire the pronunciations of characters through the grapheme-to-phoneme correspondence (GPC) rules. In the assembled-phonology training condition, participants were instructed to assemble the pronunciations of the whole characters *via* the GPC rules. They learned the pronunciations of both Hangul letters and the whole characters in the assembled-phonology training condition. The assignment of the two groups of characters into the two learning conditions was counterbalanced across participants to eliminate the effects of materials on learning. In each training session, participants spent the same time in learning the two conditions, with about half an hour for each condition.

As in previous studies (Mei et al., 2015; Li et al., 2019), several learning tasks were designed to facilitate the acquisition of the artificial language characters. The tasks included character learning (i.e., learning the character-sound associations one by one), free learning (i.e., relearning the characters that participants had difficulties), phonological choice (i.e., choosing the correct sound for the target character from four sounds), naming with feedback (i.e., reading each character aloud followed by its correct pronunciation), and fast naming (i.e., reading 10 words as fast and accurately as possible).

### fMRI task

After the 5-day artificial language training, participants were scanned once while performing an overt naming task. One group of Chinese characters and two groups artificial language characters (one group in the addressed-phonology learning and the other group in the assembled-phonology learning) were used for the fMRI task. Each group had 16 characters. Those characters were pseudo-randomly presented with the sequence was optimized by using OPTSEQ2.^1^ The inter-trial intervals were not jittered because this study used a slow event-related design which was able to precisely estimate single-trial activation patterns. There were three runs in total. Each run contained 48 trials with 16 characters in each condition presented once. Each trial lasted for 12 s. Specifically, it started with a 1 s fixation, followed by a character presented on the screen for 3 s. Participants were asked to read each word as quickly and accurately as possible. Once the character disappeared, participants were asked to perform a visual orientation judgment task for 8 s to prevent further rehearsing for the character and to keep participants undergoing the same cognitive processes after the character (Xue et al., 2013; Zhao et al., 2017; Qu et al., 2021). To make this task engaging, a self-paced procedure was used. Specifically, a Gabor image presented on the screen was tilted 45° either to the left or to the right of vertical. Participants were asked to judge the orientation of the Gabor. The Gabor image disappeared once participants responded and the next image was presented after a blank of 100 ms (see Figure 1B).

Due to the large amount of noise during scanning, participants’ oral responses were recorded in another session of the word naming task outside the scanner. The stimuli sequence and parameters used were identical with the fMRI task.

### MRI data acquisition and analysis

The fMRI data were collected using a 3.0 T Siemens MRI scanner in the MRI Center at South China Normal University. A single-shot T2*-weighted gradient-echo EPI sequence was used to collect the functional imaging data. The scanning parameters were used as follows: TR = 2,000 ms, TE = 30 ms, flip angle = 90°, FOV = 224 × 224 mm, matrix size = 64 × 64, slice thickness = 3.5 mm, and number of slices = 32. A T1-weighted, gradient-echo pulse sequence was used to collect anatomical data. The parameters were as follows: TR = 1,900 ms, TE = 2.52 ms, flip angle = 9°, FOV = 256 × 256 mm, matrix size = 256 × 256, slice thickness = 1 mm, and number of slices = 176.

The imaging data were processed using FEAT (FMRI Expert Analysis Tool) Version 6.00. The first three images in each run were discarded to allow for T1 equilibrium effects. The remaining functional images were realigned and normalized to the MNI (Montreal Neurological Institute) template (Jenkinson and Smith, 2001). They were smoothed using a 5 mm full-width at half-maximum Gaussian kernel and temporally filtered using a nonlinear high-pass filter with a 60 s cut-off. The head movement was no more than one voxel for any run or participant.

We then modeled the data using the general linear model. In each run, there were 48 separate regressors corresponding to 48 characters. The fixation and the orientation task were not explicitly modeled and served as implicit baseline. A canonical hemodynamic response function (double-gamma) was used to convolve with the onsets and durations of events. Following previous studies (Xue et al., 2010; Mumford et al., 2012; Dong et al., 2021), the single-trial neural response was estimated using least squares estimation and ridge regression. Six motion parameters were used as covariates to improve statistical sensitivity. Contrast images of the three conditions (i.e., Chinese characters, addressed characters, and assembled characters) and their comparisons were calculated for each participant and each run.

We then used a fixed-effects model to concatenate data from the three runs for each participant. After that, a random-effects model with FLAME Stage 1 was used to obtain group activations. All reported results were thresholded with a height threshold of *Z* > 2.6 (i.e., *p* < 0.005) and a cluster probability of *p* < 0.05, and the Gaussian random field theory was used to perform multiple comparison corrections for the whole brain (Worsley, 2010).

### Multivariate pattern analysis

To investigate differences in activation pattern between assembled and addressed characters, we conducted the multivariate pattern analysis (i.e., MVPA) using the CoSMoMVPA.^2^ Beta-estimates of unsmoothed data in each run were extracted and then were used to train and test the classifiers (Mur et al., 2009; Pereira et al., 2009). To implement all classifications, a linear support vector machine (SVM) classifier with a regularization parameter (c = 1) was used. A leave-one-run-out cross-validation protocol was used to calculate the classification accuracy. Specifically, we trained a classifier to distinguish characters in addressed-phonology learning versus those in assembled-phonology learning in two runs, and then tested the classifier’s ability in the left-out run. The classification accuracies were calculated as the means of the three classification results.

In the above analysis, a searchlight-based method was used. Specifically, a spherical searchlight comprised 100 voxels was moved within the whole brain. The classification accuracy (proportion of correctly classified trials) was mapped back upon the sphere’s central voxel to produce accuracy maps. The analysis was conducted in each participant’s native space and then transformed into standard space to form a 4-D map. Finally, a t-map was generated at the group level to test whether the classification accuracy of each voxel was significantly higher than the chance level (0.5). Based on the performance of those predictions, we could find out which brain regions showed differences in neural pattern between assembled and addressed characters. The group images were thresholded at *p* < 0.005 (one-tailed), and a cluster probability of *p* < 0.05.

### Correlation analysis

**Figure 3:**
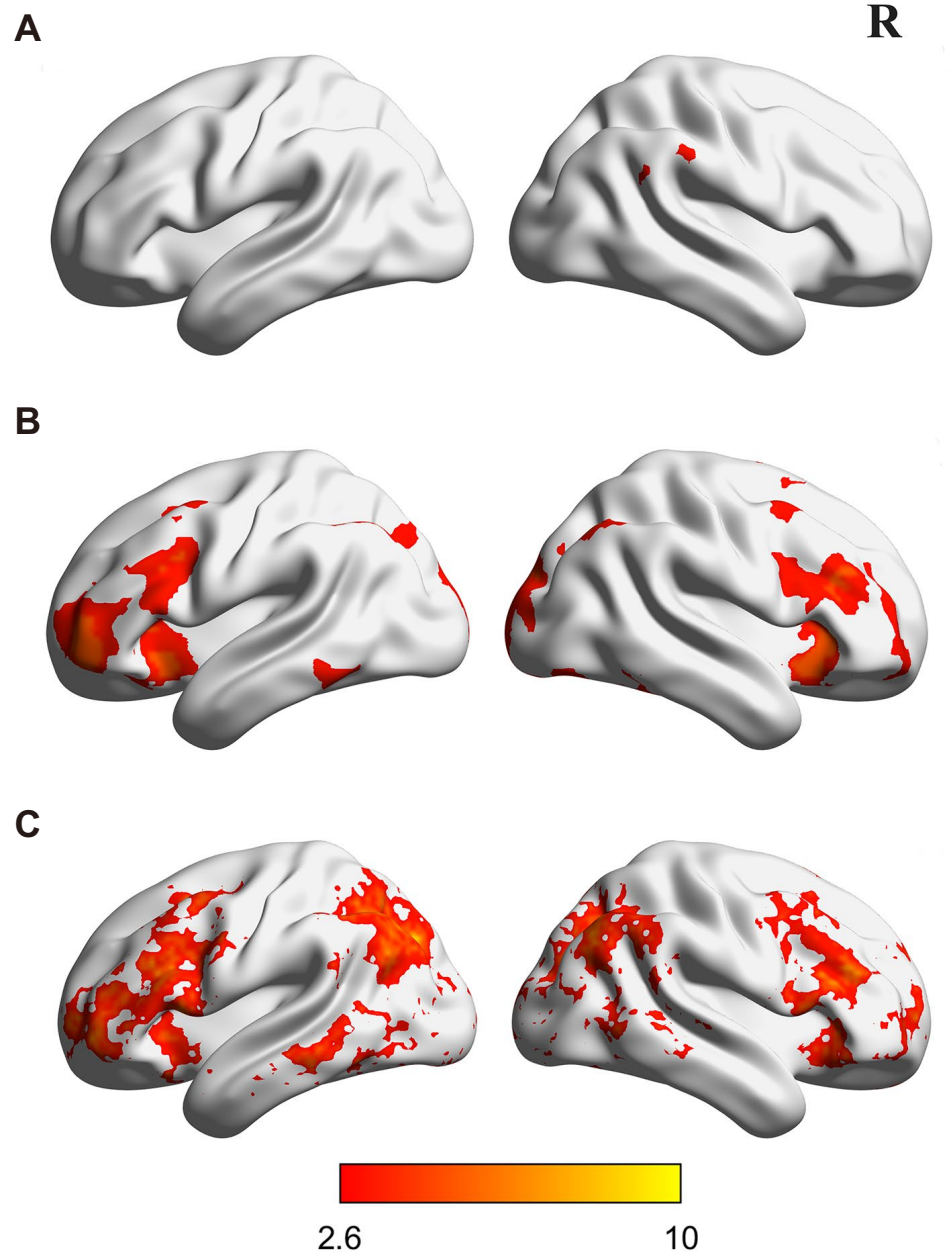
Brain regions showing different activations **(A,B)** and multivoxel activation patterns **(C)** across the two types of characters (i.e., assembled and addressed characters). All activations were thresholded at *p* < 0.005 (whole-brain corrected). R, right.

To examine individual differences in the recruitment of the two pathways for phonological access (i.e., assembled and addressed characters), we correlated activation differences between assembled and addressed characters in the regions identified in MVPA with reading strategy index. Specifically, the abovementioned MVPA revealed nine brain regions showing different neural codes between assembled and addressed characters. The brain regions included the paracingulate gyrus (PCG) and posterior cingulate gyrus (PCC) in the middle line of the brain, the bilateral pars trianguris (PT), orbitofrontal cortex (OFC), middle temporal gyrus (MTG), and the left precuneus cortex (PCun; see Figure 3C). These nine brain regions were functionally defined as regions of interest (ROI) in the correlation analysis. Each ROI was defined as a 6 mm diameter sphere around the local maxima in each cluster. In each ROI, percent signal changes were extracted separately for assembled and addressed characters (Mumford, 2007). We then correlated activation difference between addressed and assembled conditions (i.e., percent signal changes of the addressed condition minus those of the assembled condition) in each ROI with reading strategy index. This analysis would help to reveal the associations between individuals’ preferred reading strategies and the involvement of a certain region in phonological access.

To explore the effects of individuals’ preferred reading strategies on learning, we additionally calculated the correlations between reading strategy index and behavioral differences between addressed and assembled characters (i.e., difference in Z scores of reaction time or accuracy between assembled and addressed characters) during the word naming task after the fMRI scan. The Z scores were separately calculated for the addressed and assembled characters. An individual with greater difference in Z scores of accuracy or with smaller difference in Z scores of reaction time indicates that he is better at addressed-phonology learning relative to assembled-phonology learning than other individuals. Therefore, if individuals’ reading pathway preference affects phonological learning in a new language, reading strategy index would positively correlate with difference in Z scores of accuracy, but negatively correlate with difference in Z scores of reaction time.

## Results

### Behavioral results

One-way repeated measures ANOVA was used to examine behavioral differences in reaction time and accuracy (see Figure 1C). The main effects of word type were significant for both reaction time (*F*(2, 48) = 328.62, *p* < 0.001) and accuracy (*F*(2, 48) = 40.10, *p* < 0.001). *Post hoc* comparisons showed that Chinese characters (797.51 ms) were named faster than assembled characters (1402.77 ms; *p* < 0.001), which were named faster than addressed characters (1548.13 ms; *p* < 0.001). Consistently, Chinese characters (100.00%) showed higher accuracy than assembled characters (97.92%; *p* < 0.001), which showed higher accuracy than addressed characters (90.92%; *p* < 0.001). These results indicate that participants were more familiar with characters in their native language (i.e., Chinese characters) than newly-acquired characters (i.e., assembled and addressed characters). Furthermore, participants performed better on assembled characters relative to addressed characters as the former followed GPC rules. This result is consistent with previous findings that assembled phonology is easier to learn than addressed phonology even for Chinese speakers with much experience on addressed phonology (Hooper, 2001; Aro and Wimmer, 2003; Ellis et al., 2004; Mei et al., 2014, 2015).

To examine the effects of individuals’ preferred reading strategies on character learning, we further calculated the correlations between reading strategy index and behavioral differences across the two types of characters (i.e., addressed and assembled characters) after scan. Results showed that reading strategy index was significantly correlated with differences in accuracy between the two types of characters (*r* = 0.419, *p* = 0.037; see Figure 2B). The correlation for reaction time was not significant (*r* = 0.031, *p* = 0.884; see Figure 2C). These results indicate that the more lexical pathways participants preferred, the better they performed on newly-acquired addressed characters relative to assembled characters.

### Neural activations during the character naming task

**Figure 4:**
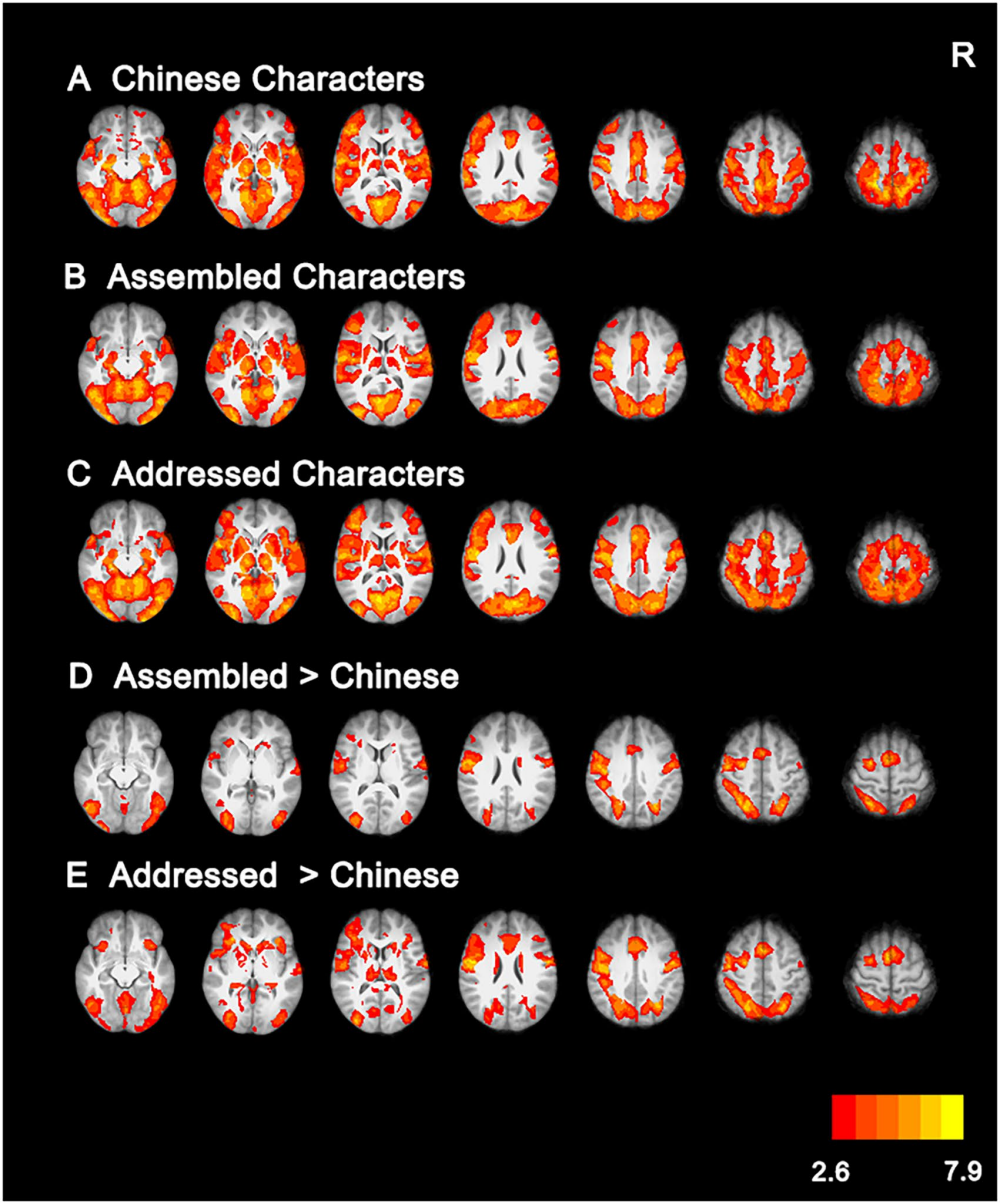
Brain activations for Chinese characters **(A)**, Assembled characters **(B)**, Addressed characters **(C)**, and their comparisons **(D,E)**. All activations were thresholded at *p* < 0.005 (whole-brain corrected). R, right.

Whole-brain activation analysis showed that Chinese characters, addressed characters, and assembled characters had similar activations in an extensive network, including the anterior cingulate cortex, supplementary motor cortex, bilateral prefrontal cortex, occipitoparietal cortex, and occipitotemporal cortex (see Figures 4A–C; Supplementary Table S1). Moreover, direct comparisons between Chinese characters and artificial language characters revealed that, compared with Chinese characters, assembled characters elicited greater activations in the bilateral precentral gyrus, temporoparietal cortex, fusiform gyrus and left inferior frontal gyrus (see Figure 4D; Supplementary Table S2), and addressed characters elicited stronger activations in the paracingulate gyrus, bilateral middle frontal gyrus, fusiform gyrus and left supramarginal gyrus (see Figure 4E; Supplementary Table S2).

Further comparisons between the two types of artificial language characters showed that assembled characters induced greater activations in the right supramarginal gyrus (see Figure 3A; Supplementary Table S3), whereas addressed characters evoked stronger activations in the paracingulate gyrus, bilateral middle frontal gyrus, inferior frontal gyrus, orbitofrontal cortex, precuneus cortex, lingual gyrus and fusiform gyrus (see Figure 3B; Supplementary Table S3).

### Brain regions showing differential activation patterns between assembled and addressed characters

A searchlight-based MVPA was used to investigate the differences in activation pattern between assembled and addressed characters. In this analysis, we trained and tested classifiers to distinguish assembled versus addressed characters. Results showed that nine brain regions showed significantly higher classification accuracy than the chance level (i.e., 50%), including the paracingulate gyrus (PCG), posterior cingulate gyrus (PCC), bilateral pars trianguris (PT), orbitofrontal cortex (OFC), middle temporal gyrus (MTG), and the left precuneus cortex (PCun; see Figure 3C; Supplementary Table S4). These results are generally consistent with brain regions reported in the activation analysis above.

### The involvement of brain regions in phonological access was modulated by individuals’ preferred reading strategies

**Figure 5:**
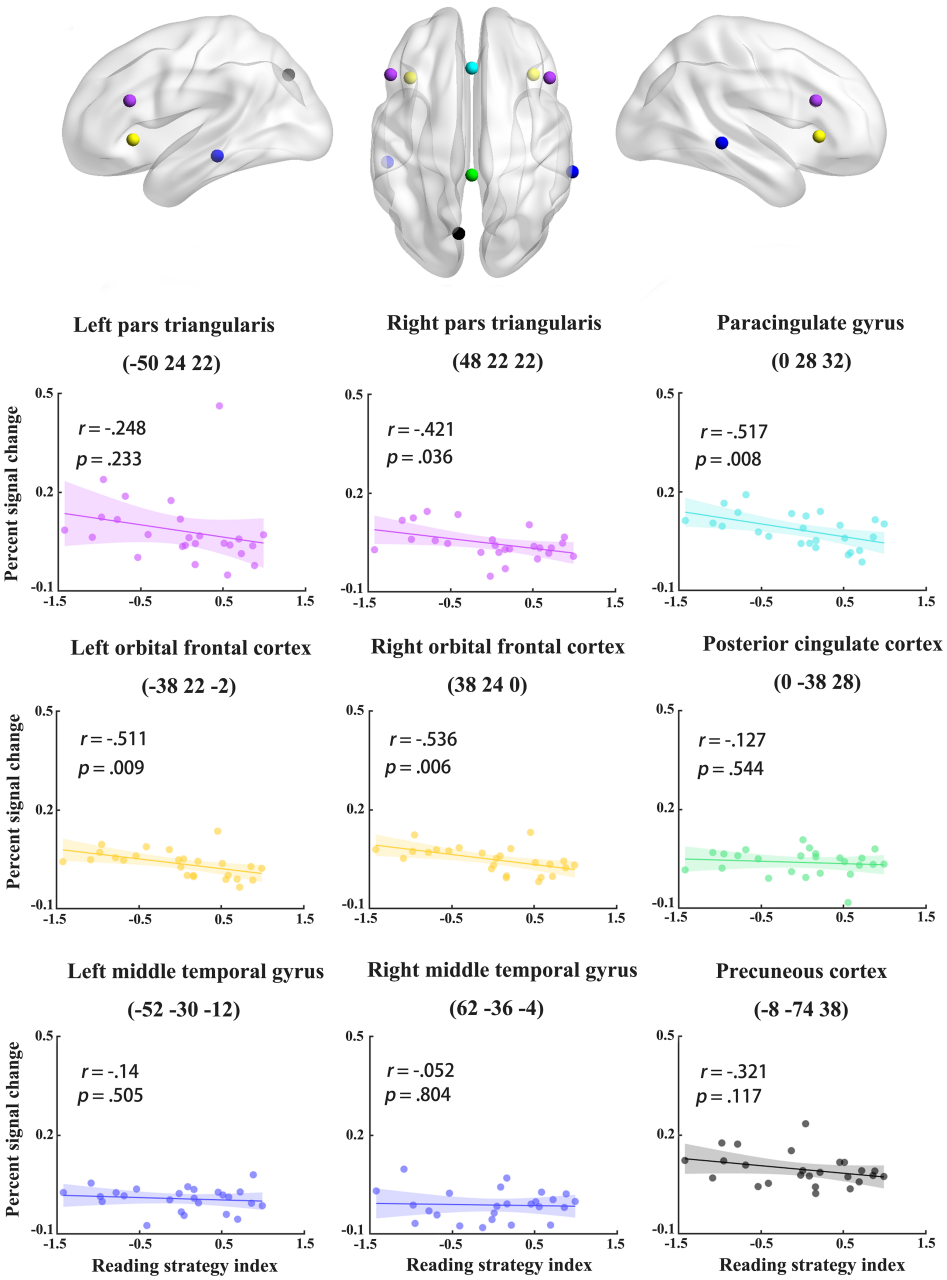
The correlations between reading strategy index and activation differences between addressed and assembled characters in the nine regions identified in multivariate pattern analysis.

To examine the associations between individuals’ preferred reading strategies and the involvement of a certain region in phonological access, we conducted correlation analysis on reading strategy index and the activation differences between addressed (relying on whole-word mapping) and assembled (relying on grapheme-to-phoneme mapping) characters in the nine regions identified in MVPA. The activation differences between addressed and assembled characters were computed by subtracting percent signal changes of assembled characters from those of addressed characters. Significant correlations were found in 4 regions, including the PCG (*r* = −0.52, *p* < 0.01), bilateral OFC (left: *r* = −0.51, *p* < 0.01; right: *r* = −0.54, *p* < 0.01), and right PT (*r* = −0.42, *p* < 0.05; see Figure 5). The correlation coefficients remained significant in the PCG (*p* = 0.027) and bilateral OFC (left: *p* = 0.027; right: *p* = 0.036) after false discovery rate (FDR) correction (Benjamini and Hochberg, 1995; Qu et al., 2019). It is worth noting that there is an outlier value in the left PT. After excluding that outlier, the correlation coefficients became significant (*r* = −0.42, *p* < 0.05). These results indicate that participants who preferred lexical pathway in phonological access show less involvement of brain regions for addressed phonology in the processing of newly-acquired addressed characters.

**Figure 6:**
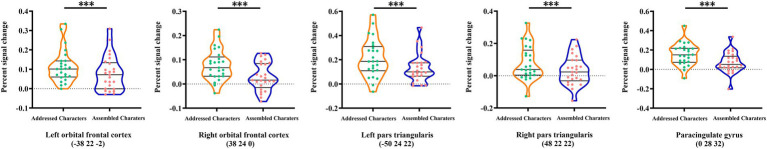
Activation differences between addressed and assembled characters in the five brain regions showing significant brain-behavior correlation. ***p < 0.001.

The involvement of the above five brain regions (i.e., PCG, bilateral OFC, and PT) in addressed phonology were further confirmed by comparison between activations of addressed and assembled characters. Results showed that all the regions showed greater activations for addressed characters than assembled character [the left OFC: *t*(24) = 4.44, *p* < 0.001; the right OFC: *t*(24) = 6.11, *p* < 0.001; the left PT: *t*(24) = 3.93, *p* < 0.01; the right PT: *t*(24) = 4.78, *p* < 0.001; and the PCG: *t*(24) = 7.93, *p* < 0.001] (see Figure 6).

## Discussion

Using an artificial language paradigm, this study examined the effects of reading pathway preference on the neural correlates of phonological learning. Behavioral results showed that the more lexical pathways participants preferred, the better they performed on newly-acquired addressed characters relative to assembled characters. Neuroimaging results showed that participants who preferred lexical pathway in phonological access showed less involvement of ventral regions (e.g., the bilateral OFC and PT) for addressed characters relative to assembled characters. These results suggest that individuals’ preference on reading pathways influence the recruitment of neural pathways in phonological learning.

As mentioned in Introduction, much research has examined the neural pathways of phonological access by contrasting natural language materials varying in reliance on the lexical or nonlexical routes (Jobard et al., 2003; Mechelli et al., 2005; Tan et al., 2005; Cattinelli et al., 2013; Cao et al., 2017; Dong et al., 2020). They found that phonological access *via* lexical route relied on regions related to semantic processing such as the ventral part of the left IFG, lateral temporal cortex, and angular gyrus (Binder et al., 2009; Price, 2012; Taylor et al., 2013), whereas that *via* nonlexical route depended on regions related to phonological processing such as the dorsal part of the left IFG, precentral gyrus, and supramarginal gyrus (Hartwigsen et al., 2010; Sliwinska et al., 2012; Mei et al., 2014). These findings were confirmed by studies using well-designed artificial language training paradigms (e.g., Mei et al., 2014, 2015). Consistent with those studies, the present study also found greater activations for addressed characters in the bilateral OFC and PT, but greater activations for assembled characters in the right supramarginal gyrus. These results were further confirmed by multivariate pattern analysis. It should be noted that our study revealed several brain regions in the right hemisphere (e.g., the right OFC and PT) showing different activations between addressed and assembled phonologies. The involvement of regions in the right hemisphere in phonological access might reflect that regions in the right hemisphere served as a complement to their left homologues to support the processing of newly-acquired words because reading non-proficient words costs relatively high cognitive resources. Consistent with this explanation, previous studies have revealed heavy involvement of regions in the right hemisphere in novel word learning or in word reading in less proficient language (Garavan et al., 1999; Chen et al., 2009; Mei et al., 2014; Kim et al., 2016; Cao et al., 2017).

More importantly, the present study provided direct neuroimaging evidence for the effects of individuals’ reading pathway preference on the involvement of the two neural pathways in phonological learning. In this study, we quantified individuals’ reading pathway preference by calculating the reading strategy index. Consistent with previous studies (Pecini et al., 2008; Ihnen et al., 2013; Mei et al., 2015), we found that individuals differed in their preference on the two reading routes (i.e., the lexical and nonlexical routes). Interestingly, inter-subject variance on reading pathway preference/strategy affected the involvement of a certain region for phonological access in phonological learning. Specifically, participants who preferred lexical pathway in phonological access showed less involvement of regions for the lexical routes (e.g., the bilateral OFC, and PT) in reading addressed characters relative to assembled characters. Negative correlations between neural activations and behavioral performance have also been reported in a number of studies (Boivin et al., 1992; Reichle et al., 2000; Newman et al., 2003; Prat et al., 2007). Less activation for individuals with better performance is thought to reflect that information is processed in a more efficient and less neural energy consuming way in their brain (Haier et al., 1992; Prat et al., 2007, 2011; Neubauer and Fink, 2009). From this perspective, our results might reflect that phonological access *via* the preferred pathway consumed less neural energy probably because of its higher efficiency in phonological access. This explanation is consistent with previous findings of less activation for the processing of more proficient language relative to less proficient language (Yetkin et al., 1996; Wartenburger, 2003; Oliver et al., 2017) and for high-frequence words relative to low-frequence ones (Kuo et al., 2003; Lee et al., 2004; Carreiras et al., 2006).

In addition, our results observed the associations between reading pathway preference and learning performance. Specifically, we found that the more lexical pathways participants preferred, the better they performed on the newly-acquired addressed characters relative to assembled characters. This result indicates that phonological learning *via* one of the two reading routes is more efficient for individuals who preferred the corresponding reading pathway or strategy. Taken together behavioral and neuroimaging results, we can conclude that individuals’ prior reading pathway preference modulates the involvement of a certain brain region for phonological access in phonological learning, and consequently affects their behavioral performance on phonological access.

It should be noted that this study revealed the modulatory effects of prior reading pathway preference on the involvement of brain regions for the lexical route but not of those for the nonlexical route in phonological learning. These results could be accounted by the dominance of the lexical route in native Chinese speakers. Specifically, as Chinese does not have GPC rules, phonologies of Chinese characters are solely accessed *via* the lexical pathway (Chen et al., 2009; Mei et al., 2015; Cao et al., 2017). In support of this view, previous neuroimaging studies on native Chinese speakers have revealed the great involvement of brain regions for the lexical route (e.g., the left middle frontal gyrus, inferior frontal gyrus, middle temporal gyrus) in phonological access, but less consistent results have been found for the nonlexical route (Tham et al., 2005; Cao et al., 2013). There is also evidence that, compared with native English speakers, native Chinese speakers recruited more brain regions for the lexical route to learn phonologies of addressed characters, but less brain regions for the nonlexical route to learn phonologies of assembled characters (Mei et al., 2015).

In sum, this study revealed remarkable inter-subject variations in reading pathway preference. Furthermore, using an artificial language paradigm, we found that prior reading pathway preference modulates the involvement of select brain regions for phonological access in phonological learning, and consequently affects the learning outcomes. These results provide direct neuroimaging evidence for the influence of reading pathway preference on phonological learning.

## Data availability statement

The original contributions presented in the study are included in the article/Supplementary material, further inquiries can be directed to the corresponding author.

## Ethics statement

The studies involving human participants were reviewed and approved by the IRB at the South China Normal University. The patients/participants provided their written informed consent to participate in this study. Written informed consent was obtained from the individual(s) for the publication of any potentially identifiable images or data included in this article.

## Author contributions

JD: data curation, formal analysis, software, visualization, writing-original draft, and writing–review and editing. QY: data curation and writing-original draft. AL: data curation, writing–original draft, and writing–review and editing. LG: writing–original draft and writing–review and editing. XS: writing–original draft and writing–review and editing. QC: supervision, writing–original draft, and writing–review and editing. LM: conceptualization, funding acquisition, methodology, supervision, writing–original draft, and writing–review and editing. All authors contributed to the article and approved the submitted version.

## Funding

This work was supported by the National Natural Science Foundation of China (31970983 and 32271098), the Guangdong Basic and Applied Basic Research Foundation (2022A1515011082), and the Foundation for Innovation Teams in Guangdong Higher Education (2017WCXTD002).

## Conflict of interest

The authors declare that the research was conducted in the absence of any commercial or financial relationships that could be construed as a potential conflict of interest.

## Publisher’s note

All claims expressed in this article are solely those of the authors and do not necessarily represent those of their affiliated organizations, or those of the publisher, the editors and the reviewers. Any product that may be evaluated in this article, or claim that may be made by its manufacturer, is not guaranteed or endorsed by the publisher.

## Supplementary material

The Supplementary material for this article can be found online at: https://www.frontiersin.org/articles/10.3389/fpsyg.2022.1067561/full#supplementary-material

## Footnotes

^1^ https://surfer.nmr.mgh.harvard.edu/optseq/

^2^ http://www.cosmomvpa.org/

## References

[ref1] Aro M. Wimmer H. (2003). Learning to read: English in comparison to six more regular orthographies. Appl. Psycholinguist. 24, 621–635. doi: 10.1017/S0142716403000316

[ref2] Benjamini Y. Hochberg Y. (1995). Controlling the false discovery rate: a practical and powerful approach to multiple testing. J. R. Stat. Soc. Ser. B: Methodol. 57, 289–300. doi: 10.1111/j.2517-6161.1995.tb02031.x

[ref3] Berndt R. S. Reggia J. A. Mitchum C. C. (1987). Empirically derived probabilities for grapheme-to-phoneme correspondences in English. Behav. Res. Methods Instrum. Comput. 19, 1–9. doi: 10.3758/BF03207663

[ref4] Binder J. R. Desai R. H. Graves W. W. Conant L. L. (2009). Where is the semantic system? A critical review and meta-analysis of 120 functional neuroimaging studies. Cereb. Cortex 19, 2767–2796. doi: 10.1093/cercor/bhp055, PMID: 19329570 PMC2774390

[ref5] Boivin M. J. Giordani B. Berent S. Amato D. A. Lehtinen S. Koepp R. A.. (1992). Verbal fluency and positron emission tomographic mapping of regional cerebral glucose metabolism. Cortex 28, 231–239. doi: 10.1016/s0010-9452(13)80051-2, PMID: 1499309

[ref6] Borghesani V. Hinkley L. B. N. Ranasinghe K. G. Thompson M. M. C. Shwe W. Mizuiri D.. (2020). Taking the sublexical route: brain dynamics of reading in the semantic variant of primary progressive aphasia. Brain 143, 2545–2560. doi: 10.1093/brain/awaa212, PMID: 32789455 PMC7447517

[ref7] Cai Q. Brysbaert M. (2010). SUBTLEX-CH: Chinese word and character frequencies based on film subtitles. PLoS One 5:e10729. doi: 10.1371/journal.pone.0010729, PMID: 20532192 PMC2880003

[ref8] Cao F. Sussman B. L. Rios V. Yan X. Wang Z. Spray G. J.. (2017). Different mechanisms in learning different second languages: evidence from English speakers learning Chinese and Spanish. NeuroImage 148, 284–295. doi: 10.1016/j.neuroimage.2017.01.042, PMID: 28110086

[ref9] Cao F. Tao R. Liu L. Perfetti C. A. Booth J. R. (2013). High proficiency in a second language is characterized by greater involvement of the first language network: evidence from Chinese learners of English. J. Cogn. Neurosci. 25, 1649–1663. doi: 10.1162/jocn_a_00414, PMID: 23654223 PMC3979436

[ref10] Carreiras M. Mechelli A. Price C. J. (2006). Effect of word and syllable frequency on activation during lexical decision and reading aloud. Hum. Brain Mapp. 27, 963–972. doi: 10.1002/hbm.20236, PMID: 16628608 PMC3261381

[ref11] Cattinelli I. Borghese N. A. Gallucci M. Paulesu E. (2013). Reading the reading brain: a new meta-analysis of functional imaging data on reading. J. Neurolinguistics 26, 214–238. doi: 10.1016/j.jneuroling.2012.08.001

[ref12] Chen C. Xue G. Mei L. Chen C. Dong Q. (2009). Cultural neurolinguistics. Prog. Brain Res. 178, 159–171. doi: 10.1016/S0079-6123(09)17811-1, PMID: 19874968 PMC2821076

[ref13] Cohen J. (1988). Statistical power analysis for the behavioral sciences. Technometrics 31, 499–500. doi: 10.1080/00401706.1989.10488618

[ref14] Cohen J. (1992). Quantitative methods in psychology: a power primer. Psychol. Bull. 112, 155–159. doi: 10.1037/0033-2909.112.1.155, PMID: 19565683

[ref15] Coltheart M. Rastle K. Perry C. Langdon R. Ziegler J. (2001). DRC: a dual route cascaded model of visual word recognition and reading aloud. Psychol. Rev. 108, 204–256. doi: 10.1037/0033-295X.108.1.204, PMID: 11212628

[ref16] Cummine J. Gould L. Zhou C. Hrybouski S. Siddiqi Z. Chouinard B.. (2013). Manipulating instructions strategically affects reliance on the ventral-lexical reading stream: converging evidence from neuroimaging and reaction time. Brain Lang. 125, 203–214. doi: 10.1016/j.bandl.2012.04.009, PMID: 22632813

[ref17] Démonet J. F. Chollet F. Ramsay S. Cardebat D. Nespoulous J. L. Wise R.. (1992). The anatomy of phonological and semantic processing in normal subjects. Brain 115, 1753–1768. doi: 10.1093/brain/115.6.1753, PMID: 1486459

[ref18] Destoky F. Bertels J. Niesen M. Wens V. Vander Ghinst M. Leybaert J.. (2020). Cortical tracking of speech in noise accounts for reading strategies in children. PLoS Biol. 18:e3000840. doi: 10.1371/journal.pbio.3000840, PMID: 32845876 PMC7478533

[ref19] Dong J. Li A. Chen C. Qu J. Jiang N. Sun Y.. (2021). Language distance in orthographic transparency affects cross-language pattern similarity between native and non-native languages. Hum. Brain Mapp. 42, 893–907. doi: 10.1002/hbm.25266, PMID: 33112483 PMC7856648

[ref20] Dong J. Lu C. Chen C. Li H. Liu X. Mei L. (2020). Functional dissociations of the left anterior and posterior Occipitotemporal cortex for semantic and non-semantic phonological access. Neuroscience 430, 94–104. doi: 10.1016/j.neuroscience.2020.01.024, PMID: 32032670

[ref21] Ellis N. C. Natsume M. Stavropoulou K. Hoxhallari L. Daal V. H. Polyzoe N.. (2004). The effects of orthographic depth on learning to read alphabetic, syllabic, and logographic scripts. Read. Res. Q. 39, 438–468. doi: 10.1598/rrq.39.4.5

[ref22] Garavan H. Ross T. J. Stein E. A. (1999). Right hemispheric dominance of inhibitory control: an event-related functional mri study. Proc. Natl. Acad. Sci. U. S. A. 96, 8301–8306. doi: 10.1073/pnas.96.14.8301, PMID: 10393989 PMC22229

[ref23] Gontijo P. F. D. Gontijo I. Shillcock R. (2003). Grapheme-phonemeprobabilities in British English. Behav. Res. Methods Instrum. Comput. 35, 136–157. doi: 10.3758/bf03195506, PMID: 12723789

[ref24] Haier R. J. Siegel B. Tang C. Abel L. Buchsbaum M. S. (1992). Intelligence and changes in regional cerebral glucose metabolic rate following learning. Intelligence 16, 415–426. doi: 10.1016/0160-2896(92)90018-M

[ref25] Harm M. W. Seidenberg M. S. (2004). Computing the meanings of words in reading: cooperative division of labor between visual and phonological processes. Psychol. Rev. 111, 662–720. doi: 10.1037/0033-295X.111.3.662, PMID: 15250780

[ref26] Hartwigsen G. Baumgaertner A. Price C. J. Koehnke M. Ulmer S. Siebner H. R. (2010). Phonological decisions require both the left and right supramarginal gyri. Proc. Natl. Acad. Sci. U. S. A. 107, 16494–16499. doi: 10.1073/pnas.1008121107, PMID: 20807747 PMC2944751

[ref27] Hoffman P. Lambon Ralph M. A. Woollams A. M. (2015). Triangulation of the neurocomputational architecture underpinning reading aloud. Proc. Natl. Acad. Sci. U. S. A. 112, E3719–E3728. doi: 10.1073/pnas.1502032112, PMID: 26124121 PMC4507229

[ref28] Hooper A. M. (2001). Why learning to read is easier in welsh than in english: orthographic transparency effects evinced with frequency-matched tests. Appl. Psycholinguist. 22, 571–599. doi: 10.1017/S0142716401004052

[ref29] Ihnen S. K. Z. Petersen S. E. Schlaggar B. L. (2013). Separable roles for attentional control sub-Systems in Reading Tasks: a combined behavioral and fMRI study. Cereb. Cortex 25, 1198–1218. doi: 10.1093/cercor/bht313, PMID: 24275830 PMC4397571

[ref30] Jamal N. I. Piche A. W. Napoliello E. M. Perfetti C. A. Eden G. F. (2012). Neural basis of single-word reading in Spanish-English bilinguals. Hum. Brain Mapp. 33, 235–245. doi: 10.1002/hbm.21208, PMID: 21391265 PMC6870354

[ref31] Jenkinson M. Smith S. (2001). A global optimisation method for robust affine registration of brain images. Med. Image Anal. 5, 143–156. doi: 10.1016/s1361-8415(01)00036-6, PMID: 11516708

[ref32] Jobard G. Crivello F. Tzourio-Mazoyer N. (2003). Evaluation of the dual route theory of reading: a metanalysis of 35 neuroimaging studies. NeuroImage 20, 693–712. doi: 10.1016/S1053-8119(03)00343-4, PMID: 14568445

[ref33] Kachlicka M. Saito K. Tierney A. (2019). Successful second language learning is tied to robust domain-general auditory processing and stable neural representation of sound. Brain Lang. 192, 15–24. doi: 10.1016/j.bandl.2019.02.004, PMID: 30831377

[ref34] Kim S. Y. Qi T. Feng X. Ding G. Liu L. Cao F. (2016). How does language distance between L1 and L2 affect the L2 brain network? An fMRI study of Korean-Chinese-English trilinguals. NeuroImage 129, 25–39. doi: 10.1016/j.neuroimage.2015.11.068, PMID: 26673115

[ref35] Kuo W. J. Yeh T. C. Lee C. Y. Wu U. Chou C. C. Ho L. T.. (2003). Frequency effects of Chinese character processing in the brain: an event-related fMRI study. NeuroImage 18, 720–730. doi: 10.1016/s1053-8119(03)00015-6, PMID: 12667849

[ref36] Lee C. Y. Tsai J. L. Kuo W. J. Yeh T. C. Wu Y. T. Ho L. T.. (2004). Neuronal correlates of consistency and frequency effects on Chinese character naming: an event-related fMRI study. NeuroImage 23, 1235–1245. doi: 10.1016/j.neuroimage.2004.07.064, PMID: 15589089

[ref37] Li H. Qu J. Chen C. Chen Y. Xue G. Zhang L.. (2019). Lexical learning in a new language leads to neural pattern similarity with word reading in native language. Hum. Brain Mapp. 40, 98–109. doi: 10.1002/hbm.24357, PMID: 30136328 PMC6865609

[ref38] Mechelli A. Crinion J. T. Long S. Friston K. J. Lambon Ralph M. A. Patterson K.. (2005). Dissociating reading processes on the basis of neuronal interactions. J. Cogn. Neurosci. 17, 1753–1765. doi: 10.1162/089892905774589190, PMID: 16269111

[ref39] Mechelli A. Gorno-Tempini M. L. Price C. J. (2003). Neuroimaging studies of word and pseudoword reading: consistencies, inconsistencies, and limitations. J. Cogn. Neurosci. 15, 260–271. doi: 10.1162/089892903321208196, PMID: 12676063

[ref40] Mei L. Xue G. Lu Z. L. He Q. Wei M. Zhang M.. (2015). Native language experience shapes neural basis of addressed and assembled phonologies. NeuroImage 114, 38–48. doi: 10.1016/j.neuroimage.2015.03.075, PMID: 25858447 PMC4446231

[ref41] Mei L. Xue G. Lu Z. L. He Q. Zhang M. Wei M.. (2014). Artificial language training reveals the neural substrates underlying addressed and assembled phonologies. PLoS One 9:e93548. doi: 10.1371/journal.pone.0093548, PMID: 24676060 PMC3968146

[ref42] Mumford J. (2007). A Guide to Calculating Percent Change with Featquery. Unpublished tech report. Available at: http://mumford.Bol.Ucla.Edu/perchange_guide.Pdf

[ref43] Mumford J. A. Turner B. O. Ashby F. G. Poldrack R. A. (2012). Deconvolving BOLD activation in event-related designs for multivoxel pattern classification analyses. NeuroImage 59, 2636–2643. doi: 10.1016/j.neuroimage.2011.08.076, PMID: 21924359 PMC3251697

[ref44] Mur M. Bandettini P. A. Kriegeskorte N. (2009). Revealing representational content with pattern-information fMRI-an introductory guide. Soc. Cogn. Affect. Neurosci. 4, 101–109. doi: 10.1093/scan/nsn044, PMID: 19151374 PMC2656880

[ref45] Neubauer A. C. Fink A. (2009). Intelligence and neural efficiency. Neurosci. Biobehav. Rev. 33, 1004–1023. doi: 10.1093/scan/nsn044 19580915

[ref46] Newman S. D. Carpenter P. A. Varma S. Just M. A. (2003). Frontal and parietal participation in problem solving in the tower of London: fMRI and computational modeling of planning and high-level perception. Neuropsychologia 41, 1668–1682. doi: 10.1016/s0028-3932(03)00091-5, PMID: 12887991

[ref47] Oliver M. Carreiras M. Paz-Alonso P. M. (2017). Functional dynamics of dorsal and ventral Reading networks in bilinguals. Cereb. Cortex 27, 5431–5443. doi: 10.1093/cercor/bhw310, PMID: 28122808

[ref48] Paulesu E. McCrory E. Fazio F. Menoncello L. Brunswick N. Cappa S. F.. (2000). A cultural effect on brain function. Nat. Neurosci. 3, 91–96. doi: 10.1038/71163 10607401

[ref49] Pecini C. Biagi L. Guzzetta A. Montanaro D. Brizzolara D. Cipriani P.. (2008). Brain representation of phonological processing in Italian: individual variability and behavioural correlates. Arch. Ital. Biol. 146, 189–203. doi: 10.1002/ana.21513, PMID: 19378881

[ref50] Pereira F. Mitchell T. Botvinick M. (2009). Machine learning classifiers and fMRI: a tutorial overview. NeuroImage 45, S199–S209. doi: 10.1016/j.neuroimage.2008.11.007, PMID: 19070668 PMC2892746

[ref51] Perry C. Ziegler J. C. Zorzi M. (2007). Nested incremental modeling in the development of computational theories: the CDP+ model of reading aloud. Psychol. Rev. 114, 273–315. doi: 10.1037/0033-295X.114.2.273, PMID: 17500628

[ref52] Plaut D. C. McClelland J. L. Seidenberg M. S. Patterson K. (1996). Understanding Normal and impaired word Reading: computational principles in quasi-regular domains. Psychol. Rev. 103, 56–115. doi: 10.1037/0033-295x.103.1.56, PMID: 8650300

[ref53] Prat C. S. Keller T. A. Just M. A. (2007). Individual differences in sentence comprehension: a functional magnetic resonance imaging investigation of syntactic and lexical processing demands. J. Cogn. Neurosci. 19, 1950–1963. doi: 10.1162/jocn.2007.19.12.1950, PMID: 17892384 PMC2599910

[ref54] Prat C. S. Mason R. A. Just M. A. (2011). Individual differences in the neural basis of causal inferencing. Brain Lang. 116, 1–13. doi: 10.1016/j.bandl.2010.08.004, PMID: 21051084 PMC3987902

[ref55] Price C. J. (2012). A review and synthesis of the first 20 years of PET and fMRI studies of heard speech, spoken language and reading. NeuroImage 62, 816–847. doi: 10.1016/j.neuroimage.2012.04.062, PMID: 22584224 PMC3398395

[ref56] Provost J.-S. Brambati S. M. Chapleau M. Wilson M. A. (2016). The effect of aging on the brain network for exception word reading. Cortex 84, 90–100. doi: 10.1016/j.cortex.2016.09.005, PMID: 27721080

[ref57] Qu J. Hu L. Liu X. Dong J. Yang R. Mei L. (2021). The contributions of the left hippocampus and bilateral inferior parietal lobule to form-meaning associative learning. Psychophysiology 58:e13834. doi: 10.1111/psyp.13834, PMID: 33949705

[ref58] Qu J. Zhang L. Chen C. Xie P. Li H. Liu X.. (2019). Cross-language pattern similarity in the bilateral fusiform cortex is associated with reading proficiency in second language. Neuroscience 410, 254–263. doi: 10.1016/j.neuroscience.2019.05.019, PMID: 31103705

[ref59] Reichle E. D. Carpenter P. A. Just M. A. (2000). The neural basis of strategy and skill in sentence picture verification. Cogn. Psychol. 40, 261–295. doi: 10.1006/cogp.2000.0733, PMID: 10888341

[ref60] Reynolds M. Besner D. Coltheart M. (2011). Reading aloud: new evidence for contextual control over the breadth of lexical activation. Mem. Cogn. 39, 1332–1347. doi: 10.3758/s13421-011-0095-y, PMID: 21830161

[ref61] Sliwinska M. W. James A. Devlin J. T. (2015). Inferior parietal lobule contributions to visual word recognition. J. Cogn. Neurosci. 27, 593–604. doi: 10.1162/jocn_a_00721, PMID: 25244114

[ref62] Sliwinska M. W. Khadilkar M. Campbell-Ratcliffe J. Quevenco F. Devlin J. T. (2012). Early and sustained supramarginal gyrus contributions to phonological processing. Front. Psychol. 3:161. doi: 10.3389/fpsyg.2012.00161, PMID: 22654779 PMC3361019

[ref63] Snyder P. J. Harris L. J. (1993). Handedness, sex, and familial sinistrality effects on spatial tasks. Cortex 29, 115–134. doi: 10.1016/s0010-9452(13)80216-x, PMID: 8472549

[ref64] Tan L. H. Laird A. R. Li K. Fox P. T. (2005). Neuroanatomical correlates of phonological processing of Chinese characters and alphabetic words: a meta-analysis. Hum. Brain Mapp. 25, 83–91. doi: 10.1002/hbm.20134, PMID: 15846817 PMC6871734

[ref65] Taylor J. S. Rastle K. Davis M. H. (2013). Can cognitive models explain brain activation during word and pseudoword reading? A meta-analysis of 36 neuroimaging studies. Psychol. Bull. 139, 766–791. doi: 10.1037/a0030266, PMID: 23046391

[ref66] Tham W. W. Rickard Liow S. J. Rajapakse J. C. Choong Leong T. Ng S. E. Lim W. E.. (2005). Phonological processing in Chinese-English bilingual biscriptals: an fMRI study. NeuroImage 28, 579–587. doi: 10.1016/j.neuroimage.2005.06.057, PMID: 16126414

[ref67] Torgesen J. Wagner R. Rashotte C. (1999). Test of word Reading efficiency (TOWRE). Novato, CA: Academic Therapy.

[ref68] Wartenburger I. (2003). Early setting of grammatical processing in the bilingual brain. Neuron 37, 159–170. doi: 10.1016/s0896-6273(02)01150-9, PMID: 12526781

[ref69] Wilson M. A. Joubert S. Ferré P. Belleville S. Ansaldo A. I. Joanette Y.. (2012). The role of the left anterior temporal lobe in exception word reading: reconciling patient and neuroimaging findings. NeuroImage 60, 2000–2007. doi: 10.1016/j.neuroimage.2012.02.009, PMID: 22361167

[ref70] Worsley K. J. (2010). Statistical Analysis of Activation Images. Available at: https://www.researchgate.net/publication/2631613_Statistical_Analysis_of_Activation_Images

[ref71] Xue G. Dong Q. Chen C. Lu Z. Mumford J. A. Poldrack R. A. (2010). Greater neural pattern similarity across repetitions is associated with better memory. Science 330, 97–101. doi: 10.1126/science.1193125, PMID: 20829453 PMC2952039

[ref72] Xue G. Dong Q. Chen C. Lu Z. L. Mumford J. A. Poldrack R. A. (2013). Complementary role of frontoparietal activity and cortical pattern similarity in successful episodic memory encoding. Cereb. Cortex 23, 1562–1571. doi: 10.1093/cercor/bhs143, PMID: 22645250 PMC3726068

[ref73] Yetkin O. Yetkin F. Z. Haughton V. M. Cox R. W. J. A. J. O. N. (1996). Use of functional MR to map language in multilingual volunteers. Am. J. Neuroradiol. 17, 473–477. doi: 10.1097/00002093-199601010-00008, PMID: 8881241 PMC8337978

[ref74] Zhao L. Chen C. Shao L. Wang Y. Xiao X. Chen C.. (2017). Orthographic and phonological representations in the fusiform cortex. Cereb. Cortex 27, 5197–5210. doi: 10.1093/cercor/bhw300, PMID: 27664959

