# Supplementary material for: Individuals’ preference on reading pathways influences the involvement of neural pathways in phonological learning
Source: Front Psychol. 2022 Dec 14;13:1067561. doi: 10.3389/fpsyg.2022.1067561 (PMC9794771; doi:10.3389/fpsyg.2022.1067561)
Supplement: Supplementary file 1 [file Table_1.DOC]

Supplementary Information

**Individuals’ Preference on Reading Pathways Influences the Involvement of Neural Pathways in Phonological Learning**

**Jie Dong^1,2,3^, Qingxin Yue^1,2,3^, Aqian Li^1,2,3^, Lala Gu^1,2,3^, Xinqi Su^1,2,3^, Qi Chen^1^, Leilei Mei^1^***

Corresponding author: Leilei Mei

Email: [**mll830925@126.com**](mailto:mll830925@126.com)

**This file includes:**

Tables S1 to S4

Table S1 Brain regions showing activations for Chinese, assembled, and addressed characters.

| **Brain regions** | **Voxels** | **x** | **y** | **z** | **Z** |
| --- | --- | --- | --- | --- | --- |
| ***Chinese characters*** |  |  |  |  |  |
| Left anterior cingulate cortex/ precuneus cortex | 2773 | -4 | 15 | 30 | 6.45 |
| Right anterior cingulate cortex/ precuneus cortex | 2967 | 7 | 10 | 32 | 5.42 |
| Left frontal pole/ inferior frontal gyrus | 3404 | -47 | 41 | 3 | 6.34 |
| Right frontal pole/ inferior frontal gyrus | 2148 | 52 | 33 | 12 | 5.22 |
| Left precentral gyrus | 3337 | -59 | -3 | 17 | 7.45 |
| Right precentral gyrus | 2786 | 57 | -1 | 14 | 6.45 |
| Left temporal pole | 525 | -50 | 5 | -4 | 6.29 |
| Right temporal pole | 653 | 54 | 18 | -13 | 5.15 |
| Left superior temporal gyrus/ middle temporal gyrus | 1912 | -48 | -25 | 0 | 6.55 |
| Right superior temporal gyrus/ middle temporal gyrus | 2496 | 65 | -25 | 3 | 5.34 |
| Left supramarginal gyrus | 1306 | -63 | -35 | 23 | 6.35 |
| Right supramarginal gyrus | 1175 | 62 | -31 | 30 | 5.26 |
| Left inferior temporal gyrus/ fusiform gyrus | 2831 | -51 | -58 | -14 | 7.49 |
| Right inferior temporal gyrus/ fusiform gyrus | 2666 | 55 | -50 | -14 | 5.31 |
| Left occipital pole/ lateral occipital cortex | 5446 | -26 | -94 | -14 | 6.41 |
| Right occipital pole/ lateral occipital cortex | 6117 | 32 | -93 | -7 | 6.37 |
|  |  |  |  |  |  |
| ***Assembled characters*** |  |  |  |  |  |
| Left anterior cingulate cortex/ supplementary motor cortex | 7280 | -3 | 12 | 30 | 6.26 |
| Right anterior cingulate cortex/ supplementary motor cortex | 1412 | 8 | 20 | 26 | 5.22 |
| Left frontal pole/ inferior frontal gyrus | 2203 | -35 | 48 | 26 | 5.02 |
| Right frontal pole/ inferior frontal gyrus | 512 | 42 | 36 | 10 | 2.89 |
| Left precentral gyrus | 3584 | -61 | -3 | 25 | 7.17 |
| Right precentral gyrus | 2662 | 57 | -3 | 25 | 7.12 |
| Left planum temporal/ superior temporal gyrus | 1270 | -61 | -12 | 6 | 6.12 |
| Right planum temporal/ superior temporal gyrus | 1410 | 65 | -20 | 8 | 5.14 |
| Left lingual gyrus/ precuneus cortex | 2569 | -3 | -71 | 6 | 7.28 |
| Right lingual gyrus/ precuneus cortex | 2871 | 6 | -67 | 6 | 6.20 |
| Left inferior temporal gyrus/ fusiform gyrus | 2062 | -49 | -59 | -16 | 7.18 |
| Right inferior temporal gyrus/ fusiform gyrus | 2038 | 47 | -56 | -16 | 5.08 |
|  |  |  |  |  |  |
| ***Addressed characters*** |  |  |  |  |  |
| Left anterior cingulate gyrus/ supplementary motor cortex | 1638 | -3 | 31 | 18 | 5.20 |
| Right anterior cingulate gyrus/ supplementary motor cortex | 1565 | 5 | 26 | 18 | 5.17 |
| Left frontal pole/ inferior frontal gyrus | 2988 | -46 | 36 | 18 | 6.23 |
| Right frontal pole/ inferior frontal gyrus | 1326 | 33 | 45 | 18 | 4.06 |
| Left planum temporal/ superior temporal gyrus | 1278 | -60 | -35 | 15 | 6.29 |
| Right planum temporal/ superior temporal gyrus | 1375 | 60 | -8 | 5 | 5.09 |
| Left precentral gyrus | 3633 | -50 | 9 | 32 | 6.26 |
| Right precentral gyrus | 2862 | 44 | -10 | 32 | 7.31 |
| Left supramarginal gyrus/ angular gyrus | 1062 | -51 | -37 | 46 | 6.32 |
| Right supramarginal gyrus/ angular gyrus | 408 | 37 | -47 | 41 | 4.14 |
| Left superior parietal lobule | 1396 | -40 | -47 | 48 | 6.17 |
| Right superior parietal lobule | 936 | 35 | -53 | 41 | 5.13 |
| Left lateral occipital cortex/ fusiform gyrus | 5824 | -35 | -84 | 6 | 7.41 |
| Right lateral occipital cortex/ fusiform gyrus | 6666 | 42 | -69 | -12 | 7.47 |
| Left lingual gyrus/ precuneus cortex | 3011 | -4 | -68 | 4 | 7.47 |
| Right lingual gyrus/ precuneus cortex | 3299 | 10 | -62 | 4 | 7.52 |

Table S2 Brain regions showing different activations between artificial language characters and Chinese characters.

| **Brain regions** | **Voxels** | **x** | **y** | **z** | **Z** |
| --- | --- | --- | --- | --- | --- |
| ***Assembled characters > Chinese characters*** |  |  |  |  |  |
| Left inferior frontal gyrus | 254 | -41 | 32 | 15 | 4.00 |
| Left precentral gyrus | 2104 | -61 | -5 | 15 | 6.10 |
| Right precentral gyrus | 709 | 59 | -3 | 15 | 5.10 |
| Right superior temporal gyrus | 267 | 66 | -15 | 1 | 4.05 |
| Left superior parietal lobule/ supramarginal gyrus | 1232 | -31 | -51 | 38 | 6.12 |
| Right superior parietal lobule/ supramarginal gyrus | 274 | 32 | -55 | 46 | 5.06 |
| Left lateral occipital cortex/ fusiform gyrus | 3146 | -49 | -64 | -12 | 6.12 |
| Right lateral occipital cortex/ fusiform gyrus | 2988 | 47 | -69 | -12 | 5.08 |
|  |  |  |  |  |  |
| ***Addressed characters > Chinese characters*** |  |  |  |  |  |
| Left paracingulate gyrus/ supplementary motor cortex | 997 | -5 | 15 | 47 | 6.16 |
| Right paracingulate gyrus/ supplementary motor cortex | 767 | 9 | 13 | 47 | 4.12 |
| Left middle frontal gyrus/ precentral gyrus | 2574 | -26 | -3 | 47 | 6.26 |
| Right middle frontal gyrus/ precentral gyrus | 981 | 40 | 29 | 24 | 4.06 |
| Left supramarginal gyrus/ superior parietal lobule | 1236 | -47 | -35 | 40 | 5.16 |
| Left lateral occipital cortex/ fusiform gyrus | 3562 | -50 | -64 | -11 | 5.14 |
| Right lateral occipital cortex/ fusiform gyrus | 3834 | 46 | -62 | -11 | 5.22 |

**Table S3 Brain regions showing different activations for assembled and addressed characters.**

| **Brain regions** | **Voxels** | **x** | **y** | **z** | **Z** |
| --- | --- | --- | --- | --- | --- |
| ***Assembled characters > Addressed characters*** |  |  |  |  |  |
| Right supramarginal gyrus/ angular gyrus | 298 | 64 | -32 | 38 | 3.73 |
|  |  |  |  |  |  |
| ***Addressed characters > Assembled characters*** |  |  |  |  |  |
| Left paracingulate gyrus/ anterior cingulate cortex | 1446 | -2 | 26 | 36 | 5.34 |
| Right paracingulate gyrus/ anterior cingulate cortex | 1361 | 4 | 25 | 36 | 5.33 |
| Left middle frontal gyrus/ inferior frontal gyrus | 1685 | -48 | 16 | 30 | 4.51 |
| Right middle frontal gyrus/ inferior frontal gyrus | 1328 | 41 | 32 | 24 | 5.38 |
| Left orbitofrontal cortex | 439 | -34 | 19 | -8 | 5.33 |
| Right orbitofrontal cortex | 440 | 28 | 22 | -8 | 4.55 |
| Left precuneus cortex/ lateral occipital cortex | 2541 | -7 | -66 | 38 | 6.27 |
| Right precuneus cortex/ lateral occipital cortex | 2488 | 12 | -75 | 38 | 6.12 |
| Left lingual gyrus/ fusiform gyrus | 1815 | -7 | -83 | -8 | 6.24 |
| Right lingual gyrus/ fusiform gyrus | 2258 | 19 | -71 | -8 | 4.61 |

Table S4 Brain regions showing different multivoxel activation patterns between assembled and addressed characters.

| **Brain regions** | **Voxels** | **x** | **y** | **z** | **Z** |
| --- | --- | --- | --- | --- | --- |
| Paracingulate gyrus | 1753 | 0 | 28 | 32 | 3.06 |
| Posterior cingulate cortex | 650 | 0 | -38 | 28 | 7.10 |
| Left pars trianguris | 454 | -50 | 24 | 22 | 7.08 |
| Right pars trianguris | 317 | 48 | 22 | 22 | 7.00 |
| Left orbitofrontal cortex | 378 | -38 | 22 | -2 | 5.09 |
| Right orbitofrontal cortex | 499 | 38 | 24 | 0 | 5.04 |
| Left middle temporal gyrus | 808 | -52 | -30 | -12 | 7.33 |
| Right middle temporal gyrus | 442 | 62 | -36 | -4 | 5.30 |
| Precuneus cortex | 1487 | -8 | -74 | 38 | 10.82 |
